# Effectiveness of isometric exercise in the management of tendinopathy: a systematic review and meta-analysis of randomised trials

**DOI:** 10.1136/bmjsem-2020-000760

**Published:** 2020-08-04

**Authors:** Christopher Clifford, Dimitris Challoumas, Lorna Paul, Grant Syme, Neal L Millar

**Affiliations:** 1 Department of Physiotherapy, NHS Greater Glasgow and Clyde, Glasgow, UK; 2 Institute of Infection, Immunity and Inflammation, College of Medicine, Veterinary and Life Sciences, University of Glasgow, Glasgow, UK; 3 Institute of Infection, Immunity and Inflammation, University of Glasgow, Glasgow, UK; 4 School of Health and Life Sciences, Glasgow Caledonian University, Glasgow, UK; 5 Department of Physiotherapy, NHS Fife, Kirkcaldy, Fife, UK

**Keywords:** tendinopathy, tendon, physiotherapy

## Abstract

**Objective:**

To systematically review and critically appraise the literature on the effectiveness of isometric exercise in comparison with other treatment strategies or no treatment in tendinopathy.

**Design:**

A systematic review and meta-analysis of randomised controlled trials.

**Data sources:**

Electronic searches of Medline, Cumulative Index to Nursing and Allied Health Literature, EMBASE and Cochrane were undertaken from inception to May 2020.

**Methods:**

Overall quality of each study was determined based on a combined assessment of internal validity, external validity and precision. For each outcome measure, level of evidence was rated based on the system by van Tulder *et al*.

**Results:**

Ten studies were identified and included in the review, including participants with patellar (n=4), rotator cuff (n=2), lateral elbow (n=2), Achilles (n=1) and gluteal (n=1) tendinopathies. Three were of good and seven were of poor overall quality. Based on limited evidence (level 3), isometric exercise was not superior to isotonic exercise for chronic tendinopathy either immediately following treatment or in the short term (≤12 weeks) for any of the investigated outcome measures. Additionally, for acute rotator cuff tendinopathy, isometric exercise appears to be no more effective than ice therapy in the short term (limited evidence; level 3).

**Summary:**

Isometric exercise does not appear to be superior to isotonic exercise in the management of chronic tendinopathy. The response to isometric exercise is variable both within and across tendinopathy populations. Isometric exercise can be used as part of a progressive loading programme as it may be beneficial for selected individuals.

**PROSPERO registration number:**

CRD42019147179.

**What is already known**

- Isometric exercise has become popular in recent years in the management of tendinopathy.
- Conflicting results have been reported in terms of immediate and short-term pain relief.
- Definitive conclusions about the effectiveness of isometric exercise in tendinopathy are yet to be made.

**What are the new findings**

- Based on the current literature, isometric exercise does not appear to be superior to isotonic exercise in the management of chronic tendinopathy.
- Isometric exercise appears to be no more effective than ice therapy in the short term for acute rotator cuff tendinopathy.
- The immediate and short-term pain response to isometric exercise is variable both within and across tendinopathy populations.
- Future research identifying which patient characteristics are more likely to affect treatment outcome and response to isometric and isotonic exercise programmes will be beneficial.

## Introduction

Tendinopathy is the preferred term for persistent tendon pain and loss of function due to mechanical loading.^1^ The burden of disease associated with tendinopathy is significant, accounting for 30% of all musculoskeletal conditions seen in general practice.^2^ It affects both sedentary^3^ and active individuals and is responsible for 30%–50% of all sporting injuries.^4^ Both the upper and lower limbs are involved, with the rotator cuff, lateral elbow, gluteal, patellar and Achilles tendons commonly affected.^4 5^

Exercise programmes are usually the first-line treatment for tendinopathy, and evidence of their effectiveness in reducing pain and improving function has been demonstrated.^6–10^ Different types of exercise or ‘loading’ programmes have been investigated, with those focusing on eccentric exercises the most commonly researched.^11–14^ However, eccentric loading has not been consistently found to be superior when compared with combined concentric/eccentric programmes.^11–14^ Although the benefits of loading programmes are well recognised, 35%–45% of individuals do not experience a significant reduction in symptoms from either eccentric or combined concentric/eccentric exercise.^15–17^ In contrast to isotonic exercise, in which the tension in the muscle remains constant despite a change in length, the muscle-tendon unit remains at a constant length during isometric exercise.^18^ Importantly however, the tendon lengthens when subjected to loading, regardless of muscle contraction type.^19^

There has been recent clinical and research interest in isometric exercise programmes in the management of tendinopathy since the study by Rio and colleagues in 2015.^20^ They reported significantly greater pain relief immediately postintervention following a single session of isometric exercise when compared with isotonic exercise in a small sample of volleyball players with patellar tendinopathy. Subsequently, it was proposed that isometric exercise be used at the start of rehabilitation to achieve a reduction in pain.^21^ A number of research groups have since investigated the effect of similar isometric loading programmes for pain relief in various tendinopathy populations and reported variable results.^22–25^

Previous systematic reviews have evaluated eccentric and combined concentric/eccentric programmes, but only one review to date has evaluated isometric exercise.^26^ This review focused on patellar tendinopathy and concluded that isometric exercise programmes appeared to be effective in short-term pain relief in athletes during the competitive season. Despite their recent popularity, it is unclear if isometric exercise provides superior pain relief when directly compared with other interventions. Definitive conclusions about the benefits of isometric exercise for tendinopathy can therefore not be made, and no previous systematic reviews have evaluated the effectiveness of isometric exercise in the management of all tendinopathies.

The aim of this systematic review of randomised clinical trials (RCTs) was to assess the effectiveness of isometric exercise in comparison with other treatment strategies or no treatment in tendinopathy. Pain was our primary outcome measure, and functional disability, range of movement (ROM), muscle strength, quality of life (QoL), satisfaction, structural integrity and cortical inhibition were secondary outcome measures.

## Methods

The present systematic review has been conducted and authored according to the Preferred Reporting Items for Systematic Reviews and Meta-Analyses (PRISMA) guidelines.^27^ The review was registered at the International Prospective Register of Systematic Reviews (PROSPERO) prior to identification of articles and data extraction.

### Eligibility

Included studies had a randomised design (of any kind) and compared isometric exercise with any treatment modality (or no treatment) for any type of tendinopathy in terms of any of the following outcomes: ‘pain’, ‘functional disability’, ‘range of movement’, ‘strength’, ‘satisfaction’, ‘quality of life’, ‘structural integrity’ and ‘cortical inhibition’. Non-randomised observational studies, case reports, case series, literature reviews and studies comparing different regimens of isometric exercise were excluded. Participants had to be 16 years of age and above with a clinical diagnosis of tendinopathy with or without radiological signs. No specific criteria were used for the diagnosis of tendinopathy; however, studies were excluded if they did not include appropriate diagnostic criteria. Studies of patients with full tendon tears or previous tendon surgery were excluded. Duration of symptoms/signs was not an exclusion criterion, neither was length of conservative treatment and follow-up. Studies were only included if published in English.

### Search strategy

A thorough literature search was conducted by two of the authors (CC and DC) independently via Medline, EMBASE, Cochrane and Cumulative Index to Nursing and Allied Health Literature from inception to May 2020, with the following Boolean operators: “(tendinopathy OR tendinosis OR tendinitis OR rotator cuff OR shoulder OR lateral elbow OR tennis elbow OR epicondylitis OR gluteal OR greater trochanteric OR patella* OR Jumper’s knee OR Achilles) AND (isometric OR static)”.

Medical Subject Headings (MeSH) terms were not used to minimise the risk of missing relevant articles. Review articles were used to identify eligible articles that were missed at the initial search.

Additionally, reference list screening and citation tracking in Google Scholar were performed for each relevant article. Screening

**Figure 1:**
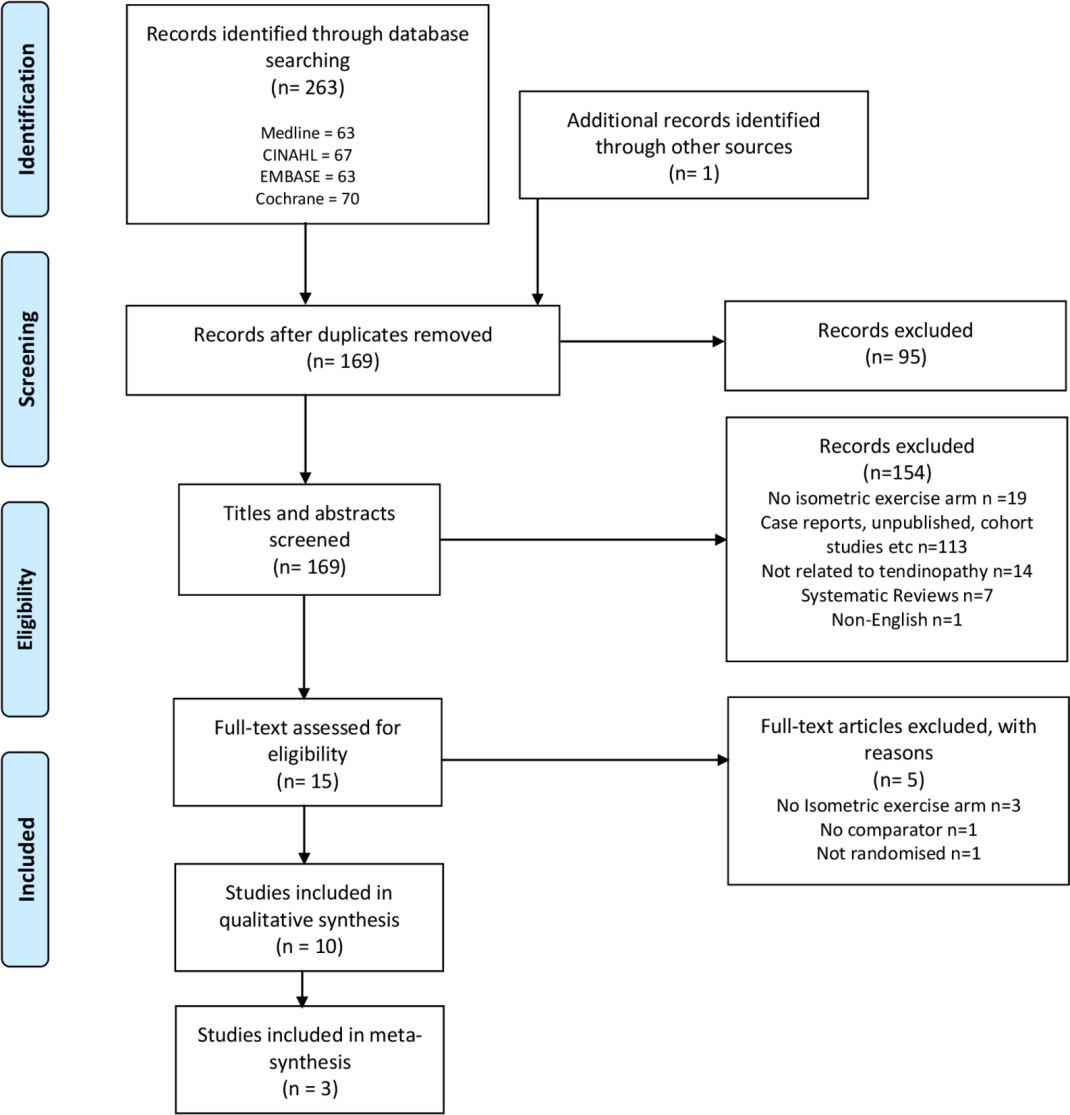
PRISMA flow diagram of included studies. CINAHL, Cumulative Index to Nursing and Allied Health Literature; PRISMA, Preferred Reporting Items for Systematic Reviews and Meta-Analyses.

A total of 264 articles were initially identified, including those from missed studies identified by review articles. After exclusion of duplicate and non-eligible articles from title and abstract screening, reference list screening and citation tracking, 10 studies were found to fulfil the eligibility criteria. Figure 1 illustrates the article screening process according to PRISMA guidelines.

### Quality assessment

For a thorough assessment of the studies, internal validity (freedom from bias), external validity (generalisability/applicability) and precision (reproducibility/freedom from random error) were all assessed separately by two of the authors (DC and CC) independently, and a third independent opinion (NLM) was sought where disagreements existed. For internal validity the ‘Cochrane Collaboration’s tool for assessing risk of bias in randomised trials’ was used on a study level (not outcome measure level), which includes seven questions/criteria (making up six categories) assessing the risk of six specific and one non-specific (‘other’) types of bias.^28^ As ‘other’ bias, our preset assessment criteria were (1) adequate and appropriate inclusion and exclusion criteria, (2) differences between treatment and control groups at baseline (confounding), (3) appropriateness of statistical tests deployed, (4) adherence of participants to assigned treatment, and (5) other methodological flaws not included in the specific categories of the tool. External validity was assessed based on the population, age range and clinical relevance of interventions and outcome measures. For the assessment of precision, performance of statistical power calculation (sample size adequate for at least 80% power) and p values that were used to define statistical significance were considered.

In the Cochrane Collaboration’s tool, each item is classified as of ‘high’, ‘low’ or ‘unclear’ risk of bias. No total scores are given. External validity and precision of each study were rated separately as of ‘high’, ‘low’ or ‘unclear’ risk.

Overall, studies were characterised as of ‘good’, ‘moderate’ or ‘poor’ quality based on a combined assessment of their internal validity, external validity and precision, which was again conducted by two of the authors independently (CC and DC) and the opinion of a third author (LP and GS) was provided where the two judgements differed. The criteria used for overall quality assessment were as follows: ‘Good’ quality studies had ‘high’ risk of bias in less than two of the internal validity categories, external validity and precision. ‘Moderate’ quality studies had ‘high’ risk of bias in two of the internal validity categories, external validity and precision. ‘Poor’ quality studies had ‘high’ risk of bias in more than two of the internal validity categories, external validity and precision.

### Data extraction: handling

Each of the eligible articles was read by the first and second authors and their key characteristics were extracted into tables to facilitate analysis and presentation. Two separate sets of tables were created by the two authors and these were subsequently compared and merged into one set to maximise accuracy of data extraction and analysis.

For the classification of strength of evidence for each outcome reported, the rating system formulated by van Tulder *et al*^29^ was used, which consists of four levels of evidence. Strong evidence (level 1) is provided by generally consistent findings in multiple high-quality RCTs. Moderate evidence (level 2) is provided by generally consistent findings in one high-quality RCT and one or more low-quality RCTs, or by generally consistent findings in multiple low-quality RCTs. Limited or conflicting evidence (level 3) is provided by only one RCT (either high or low quality) or by inconsistent findings in multiple RCTs. No evidence (level 4) is defined by the absence of RCTs. As our overall quality assessment included a ‘moderate’ quality category, we extended level 2 to ‘evidence provided by generally consistent findings in high-quality RCT and 1 or more low-quality or moderate-quality RCTs or multiple-moderate quality RCTs’. Two of the authors (DC and CC) jointly decided on the level of evidence for each outcome based on the aforementioned system without any disagreements. Results were considered to be significant when they were based on either strong or moderate evidence.

Where studies used tools and questionnaires with mixed outcome measures (eg, Victorian Institute of Sport Assessment (VISA): ‘pain’ and ‘function’), their results were tabulated under the generic outcome category ‘functional disability’. Where results of their specific subcomponents were presented too, additional results were tabulated under the corresponding outcome category (eg, pain subcomponent VISA-P score: ‘pain’).

Due to the significant heterogeneity of outcome measures used in studies, some of them were considered to represent one of our preset outcome measures as follows (according to their overall intended purpose), in order for grouping of results and hence conclusions to be possible: Global Rating of Change (GROC): ‘satisfaction’; Patient-Rated Tennis Elbow Evaluation (PRTEE): ‘functional disability’; pain-free grip strength: ‘functional disability’; Disabilities of the Arm, Shoulder and Hand (DASH): ‘functional disability’; Western Ontario Rotator Cuff Index (WORC): ‘QoL’; and Victorian Institute of Sport Assessment (VISA): ‘functional disability’.

### Statistical analysis

Where two or more studies reported results on the same comparisons and at similar follow-up time frames, the data were meta-analysed only if study participants had the same type of tendinopathy, otherwise they were only included in the qualitative analysis. An inconsistency test was conducted first (χ^2^ and I^2^ statistic), and statistical tests and forest plots were only produced if heterogeneity was no greater than 75%. The Review Manager V.5 (RevMan)^1^ software was used for statistical tests and forest plots. A random-effects meta-synthesis was employed as wide-range variability in studies’ settings was expected. For the calculation of 95% CI, where not stated by the authors, the SD was used as per the following formula:

CI=(mean_1_–mean_2_)±2√ [(SD_1_^2^/n_1_)+(SD_2_^2^/n_2_)]

When only IQR was reported, the SD was calculated as IQR/1.35. When only median was reported, mean was assumed the same as median as suggested by the *Cochrane Handbook for Systematic Reviews of Interventions* Version 5.1.0, Chapter 7.7.3.5.^30^ When CIs of means were reported, SDs were calculated by dividing the length of the CI by 3.92, and then multiplying by the square root of the sample size.^30^ Statistical significance was set at p<0.05, and all values are given at one decimal place. Publication bias was not formally assessed as the number of included studies was small.

### Deviations to protocol

According to our published protocol, results of the review would be reported at short-term (<6 weeks), mid-term (6 weeks–6 months) and long-term (>6 months) follow-up. We additionally included ‘immediate post-intervention’ results as reported by some studies as their aim was to assess for pain relief immediately after the intervention. Additionally, we extended our ‘short-term’ follow-up category to <12 weeks, which was the maximum follow-up time point in our results and also that reported as the upper limit of ‘short-term’ by most other published reviews.

## Results

Overall 10 eligible studies were identified with a total of n=294 participants. The following interventions were used: n=8 studies isolated isometric exercise, n=8 studies isolated isotonic exercise, n=2 studies combined isotonic/isometric exercise, n=2 studies ice therapy, n=1 study combined isometric exercise/ice therapy, and n=1 study no treatment (‘wait and see’). In one study where the treatment groups had either isometric exercise or ice therapy for 2 weeks, both groups subsequently had isotonic exercise for 4 weeks.^31^ Otherwise there was no overlap of treatment modalities except for the aforementioned combined groups. The mean age was 39.2 years (range 16–86).

Affected tendons by anatomical area were rotator cuff^31 32^ (n=2 studies, 63 participants), lateral elbow^33 34^ (n=2 studies, 74 participants), patellar^20 22 35 36^ (n=4 studies, 76 participants), Achilles^37^ (n=1 study, 44 participants) and gluteal^17^ (n=1 study, 30 participants). All 10 studies had a randomised design with a control group (isotonic exercise n=7 studies, ice therapy n=2 studies, no treatment n=1 study). Two studies had a cross-over design.^20 22^

Two studies included patients with acute tendinopathy (duration of symptoms ≤12 weeks), seven with chronic tendinopathy (duration of symptoms >12 weeks) and one with tendinopathy of unspecified chronicity. Treatment duration varied from a single session to 3 months and length of follow-up from 45 min to 3 months. Results were divided into (1) immediate post-treatment (three studies) and (2) short-term (≤12 weeks; seven studies). Publication years ranged from 2015 to 2020, with no RCTs published prior to 2015.

Table 1 shows the methodological characteristics, and table 2 presents a summary of samples, interventions and outcome measures of the included studies.

**Table 1:** Methodological characteristics, inclusion and exclusion criteria, and follow-up completion rates of the included studies

Author	Study type	Randomisation method	Blinding method	Allocation concealment	Statistical power calculation	Baseline comparison	Inclusion criteria	Exclusion criteria	Follow-up completion (%)
Gatz *et al*^37^	RCT.	Numbered envelopes.	None.	Sealed envelopes.	Yes; sample size adequate for 82% power.	No difference.	≥18 years, insertional or midportion Achilles tendinopathy diagnosed with history and examination (provoked pain by palpation), physical ability to perform exercises.	Pregnancy, very high (>35) or very low (<17) BMI, previous rupture or operation in area of symptoms, injections in last 6 months.	71
Clifford *et al*^17^	Pilot RCT.	Random selection of sealed, opaque envelopes from box.	None.	Sealed, opaque envelopes.	No.	No difference.	≥18 years, lateral hip pain >3 months, pain over greater trochanter and one of the following: (1) single leg stand 30 s, (2) FADER, (3) FADER and resisted IR, (4) FABER, and (5) resisted hip abduction at end-range adduction.	Physiotherapy in previous 6 months, corticosteroid injection in previous 3 months, hip or lumbar spine surgery in previous 12 months, moderate to severe hip osteoarthritis.	77
Holden *et al*^22^	RCT single-blind (cross-over).	Computer-generated allocation sequence.	Researcher blinded to sequence allocation. Participants blinded to study hypothesis.	Sealed, opaque envelopes, sequence randomised by independent researcher.	Yes; sample size adequate for 90% power.	No difference.	18–40 years of age, patellar tendinopathy clinically and radiologically.	Concurrent knee pathologies, previous knee surgery, corticosteroid injection in last 6 months.	95
Vuvan *et al*^34^	RCT.	Computer-generated.	Not stated.	Sealed, opaque envelopes.	Yes; sample size adequate for 80% power.	No difference.	18–70 years, symptoms for >6 weeks, NPRS ≥2/10, at least two of the following: (1) gripping, (2) palpation of lateral epicondyle, (3) stretching of forearm muscles, (4) resisted wrist, second or third finger extension, and (5) reduced pain-free grip strength.	Other sources of elbow pain, major neurological, inflammatory or systemic conditions, treatment by a healthcare practitioner within the previous 3 months, injections within the preceding 6 months, or major trauma, fracture or surgery in the last year.	98
Dupuis *et al*^31^	RCT single-blind.	Random number generator and block design.	Blinded outcome assessment (participants asked not to discuss their treatment with assessor).	Sealed, opaque envelopes.	Yes; sample size adequate for 80% power.	Cryotherapy group older.	16–65 years, symptoms <6 weeks, painful arc of movement, positive Neer’s or Kennedy-Hawkins and pain on resisted isometric external rotation, abduction, or positive Jobe’s all present.	Upper limb fracture, previous neck or shoulder surgery, cervical spine involvement, frozen shoulder, sign of full cuff tear, rheumatological, inflammatory or neurological disease.	77
Parle *et al*^32^	RCT single-blind pilot.	Random number generator.	Chief investigator and sonographer blinded to groups.	Sealed, opaque envelopes.	No.	Not done.	Unilateral shoulder pain <12 weeks, symptoms aggravated with active or resisted movement, unaccustomed increase in shoulder activity preceding symptoms, evidence of bursitis or tendinosis on ultrasound.	Dominant biceps pain, frozen shoulder, full thickness or large partial thickness tears, and traumatic onset of pain.	100
Rio *et al*^35^	RCT single-blind.	Random number generator function of Microsoft Excel.	Not stated.	Unmarked, opaque envelopes.	No.	No difference.	Elite and subelite volleyball and basketball athletes over 16 years of age.	Existence of other knee pathology, previous patellar tendon rupture, previous patellar tendon surgery, inflammatory disorders, metabolic bone diseases and type II diabetes, use of fluoroquinolones or corticosteroids in the last 12 months, known familial hypercholesterolaemia and fibromyalgia.	62
							Diagnosis made clinically and radiologically.		
Stasinopoulos and Stasinopoulos^33^	RCT single-blind.	‘By drawing lots’.	Outcome assessor blinded to groups.	Not stated.	No.	No difference.	Amateur tennis athletes.	Dysfunction in the shoulder, neck (radiculopathy) and/or thoracic region, local or generalised arthritis, neurological deficit, radial nerve entrapment, limitations in arm functions, the affected elbow had been operated on, had received any conservative treatment in the 4 weeks before entering the study.	100
							Clinical diagnosis of lateral elbow tendinopathy for at least 4 weeks.		
van Ark *et al*^36^	RCT single-blind.	Random number generator function of Microsoft Excel.	Not stated.	Unmarked, opaque envelopes.	No.	No difference.	Elite and subelite volleyball and basketball athletes over 16 years of age.	Existence of other knee pathology, previous patellar tendon rupture, previous patellar tendon surgery, inflammatory disorders, metabolic bone diseases and type II diabetes, use of fluoroquinolones or corticosteroids in the last 12 months, known familial hypercholesterolaemia and fibromyalgia.	62
							Diagnosis made clinically and radiologically.		
Rio *et al*^20^	RCT single-blind (cross- over).	Participants chose an envelope for order of intervention.	Not stated.	Unmarked, opaque envelopes.	No.	No difference.	Volleyball players who were taking no medications. Diagnosis made clinically and radiologically.	–	100

BMI, body mass index; FABER, flexion abduction external rotation; FADER, flexion adduction external rotation; IR, internal rotation; NPRS, Numerical Pain Rating Scale; RCT, randomised controlled trial.

**Table 2:** Samples, characteristics of interventions and outcome measures of the included studies

Author	Tendon affected	Sample, mean/median age (range); % F	Symptom duration	Interventions	Treatment duration (follow-up)	Adherence to treatment	Outcome measures
Gatz *et al*^37^	Achilles.	n=42, mean 49.5 years (21–73 years); 36%.	2–100 months (mean 27.5 months).	Isotonic (eccentric) exercise (n=20). Isotonic (eccentric) exercise + isometric exercise (n=22).	3 months (0, 1 month, 3 months).	100% in first month and around 50% thereafter.	VISA-A (pain, function). AOFAS (pain, function). Likert scale (clinical improvement with treatment). Roles and Maudsley score (clinical improvement with treatment). Tissue elasticity (shear wave elastography).
Clifford *et al*^17^	Gluteal.	n=30, mean 59.3 years (24–86 years); 90%.	Mean 23 months.	Isometric exercise (n=15). Isotonic exercise (n=15).	12 weeks (0, 4 weeks, 12 weeks).	70% of isometric group and 58% of isotonic group completed 80% of treatment sessions.	Tendon-specific: VISA-G (pain, function). NPRS 0–10 for pain. EQ-5D-5L (QoL). IPAQ-SF. GROC. PCS. HOOS.
Holden *et al*^22^	Patellar.	n=21, mean 26.5 years (18–40 years); 41%.	10–84 months (mean 24 months).	Isometric exercise (n=21). Dynamic (isotonic) exercise (n=21). Same group performed both interventions.	Single session of each intervention.	N/A.	Pain intensity during SLDS (NRS 0–10). Pressure pain thresholds. Patellar tendon thickness (USS).
Vuvan *et al*^34^	Wrist extensors.	n=40, mean 48.5 years (?– ? years); 28%.	2–8 months (mean 4 months).	Wait and see (n=19). Isometric exercise (n=21).	8 weeks (0–8 weeks).	87%.	GROC. Tendon-specific: PRTEE (pain, disability). VAS 0–10 for pain. Pain-free grip strength (function). Thermal and pressure pain threshold (nervous system sensitisation).
Dupuis *et al*^31^	Rotator cuff.	n=43, mean 38.3 years (18–65 years); 44%.	<6 weeks.	Weeks 0–2: Isometric exercise (n=20). Ice therapy (n=23). Weeks 3–6: Both groups same isotonic exercise programme.	6 weeks (0, 2 weeks, 6 weeks).	Weeks 0–2: exercise group 76% and cryotherapy group 80%. Weeks 3–6: exercise group 40% and cryotherapy group 70%.	USS to measure acromiohumeral distance. Strength (maximum force in shoulder abduction and external rotation). ROM. DASH (functional disability). Tendon-specific: WORC (QoL). BPI (pain 0–10).
Parle *et al*^32^	Rotator cuff.	n=20, mean 50 years (20–67 years); 65%.	<12 weeks.	Ice therapy (n=6). Isometric exercise (n=7). Ice therapy plus isometric exercise (n=7).	1 week (0–1 week).	Unclear.	VAS 0–10 for pain. DASH (functional disability). Strength (maximum force in shoulder flexion). Structural integrity (USS).
Rio *et al*^35^	Patellar.	n=29, mean 23 years (16–32 years); 7%.	1–120 months (mean 35.8 months).	Isometric exercise (n=13). Isotonic exercise (n=16).	4 weeks (0, daily pre- exercise and postexercise, 4 weeks).	Unclear.	Pain during the SLDS (NRS 0–10) preintervention and postintervention. Tendon-specific: VISA-P (pain, function).
Stasinopoulos and Stasinopoulos^33^	Wrist extensors.	n=34, mean 43 years; 56%.	>4 weeks (mean 6 months).	Eccentric exercise (n=11). Eccentric-concentric exercise (n=12). Eccentric-concentric exercise + isometric exercise (n=11).	4 weeks (0, 4 weeks, 8 weeks).	Unclear.	VAS 0–10 for pain. Pain-free grip strength (function). VAS 0–10 for function.
van Ark *et al*^36^	Patellar.	n=29, mean 23 years (16–32 years); 7%.	1–120 months (mean 35.8 months).	Isometric exercise (n=13). Isotonic exercise (n=16).	4 weeks (0–4 weeks).	Unclear.	Pain during the SLDS (0–10). Tendon-specific: VISA-P (pain, function).
Rio *et al*^20^	Patellar.	n=6, median 27 years (18–40 years); 0%.	Not stated.	Isometric exercise (n=6). Isotonic exercise (n=6). Same group performed both interventions.	Single session of each intervention (0–45 min).	N/A.	Pain during the SLDS (NRS 0–10). Strength (quadriceps MVIC). Measures of corticospinal excitability and inhibition.

The clinical parameters assessed by each questionnaire in the outcome measures are stated in brackets.

AOFAS, American Orthopaedic Foot and Ankle Score; BPI, Brief Pain Inventory; DASH, Disabilities of the Arm, Shoulder and Hand; EQ-5D-5L, EuroQoL 5 Dimensions 5 Level Index; GROC, Global Rating of Change; HOOS, Hip Disability and Osteoarthritis Outcome Score; IPAQ-SF, International Physical Activity Questionnaire-Short Form; MVIC, maximum voluntary isometric contraction; N/A, not applicable; NPRS, Numeric Pain Rating Scale; NRS, Numeric Rating Scale; PCS, Pain Catastrophising Scale; PRTEE, Patient-Rated Tennis Elbow Evaluation; QoL, quality of life; ROM, range of movement; SLDS, single leg decline squat; USS, ultrasound scan; VAS, Visual Analogue Scale; VISA-A, Victorian Institute of Sport Assessment-Achilles tendon; VISA-G, Victorian Institute of Sport Assessment-gluteal tendons; VISA-P, Victorian Institute of Sport Assessment-patellar tendon;; WORC, Western Ontario Rotator Cuff Index.

### Quality assessment

Table 3 illustrates our assessment of internal validity, external validity, precision and overall quality of each study. Three studies were found to be of ‘good’ overall quality and seven of ‘poor’ quality.

**Table 3:** Quality assessment of included studies (internal validity, external validity, precision and overall quality)

First author (year)	Internal validity (Cochrane Collaboration’s tool for assessing risk of bias)							External validity	Precision	Overall quality
	Selection bias		Performance bias	Detection bias	Attrition bias	Reporting bias	Other			
	Random sequence generation	Allocation concealment	Blinding of patients and staff	Blinding of outcome measures	Completeness of outcome data	Selective reporting				
Gatz (2020)^37^	Low	Low	High	High	Low	High	Low	Low	Low	Poor
Clifford (2019)^17^	Low	Low	High	High	Low	High	High	Low	High	Poor
Holden (2020)^22^	Low	Low	Low	High	Low	Low	Low	Low	Low	Good
Vuvan (2020)^34^	Low	Low	High	Low	Low	Low	Low	Low	Low	Good
Dupuis (2018)^31^	Low	Low	Low	Low	Low	Low	High	Low	Low	Good
Parle (2017)^32^	Low	Low	High	Low	Low	Low	High	Low	High	Poor
Rio (2017)^35^	Low	Low	High	High	High	Low	High	High	High	Poor
Stasinopoulos (2017)^33^	Low	?	High	Low	Low	Low	?	High	High	Poor
van Ark (2015)^36^	Low	Low	High	High	High	Low	High	High	High	Poor
Rio (2015)^20^	Low	Low	High	?	Low	Low	High	High	High	Poor

?: unclear risk of bias.

#### Internal validity

##### Selection bias

All 10 studies were randomised and were thought to have ‘low’ risk of bias for ‘random sequence generation’ (see table 1, ‘randomisation method’). Risk of bias with regard to allocation concealment was considered ‘low’ in nine studies, where the authors specifically stated that sealed, opaque envelopes were used. The study by Stasinopoulos and Stasinopoulos^33^ was classified as ‘unclear’ risk as details were not provided.

#### Performance bias

None of the studies was double-blinded due to the inherent differences between the interventions making it impossible for patients to be blinded. However, where attempts were made to minimise the risk of performance bias introduced by patients not being blinded, those studies were labelled as ‘low’ risk. In the study by Holden *et al*^22^ participants were blinded to the study hypothesis, and similarly in the study by Dupuis *et al*^31^ participants were unaware of the treatment provided to other participants.

#### Detection bias

Blinding of outcome measures was thought to be sufficient (‘low’ risk) in studies where attempts were made to blind the assessors by (1) using independent assessors and (2) asking the participants not to disclose the nature of their treatment to assessors (Holden *et al*,^22^ Dupuis *et al*,^31^ Vuvan *et al*,^34^ and Stasinopoulos and Stasinopoulos^33^). Where it was obvious that the outcome assessors were not blinded or where it was not mentioned, studies were labelled as ‘high risk’ (Parle *et al*,^32^ Clifford *et al*,^17^ Rio *et al*,^35^ van Ark *et al*,^36^ Rio *et al*,^20^ Gatz *et al*^37^).

#### Attrition bias

Rate of follow-up completion was considered of ‘high’ risk in the study by Rio *et al*^35^ and van Ark *et al*^36^ (62%). Reasons for dropouts/withdrawals of participants were adequately reported in all studies (‘low’ risk). The study by Gatz *et al*^37^ was rated as ‘low’ risk of attrition bias despite the significant loss to follow-up (25% and 32% in the two groups) as the remaining participants were sufficient for the minimum sample sizes based on their power calculation.

#### Reporting bias

Eight studies were thought to be of ‘low’ risk of bias regarding reporting of results as they included clinically relevant outcome measures, adequate graphical illustration of their results and reporting of results of statistical tests. In the study by Clifford *et al*,^17^ no p values were reported for any of the comparisons (‘high’ risk). In the study by Gatz *et al*,^37^ performance in two of the secondary outcome measures (Likert scale, Roles and Maudsley score) was not compared with statistical tests. Additionally, even though it constitutes part of the VISA-A questionnaire, no specific comparisons were carried out for pain, which is considered an important clinical symptom (‘high’ risk).

#### Other bias

Inclusion and exclusion criteria were thought to be adequate for all but two studies: Rio *et al*^20^ did not use any exclusion criteria, and the exclusion criteria in Parle *et al*^32^ were very limited. Comparison of baseline characteristics of the treatment groups was reported by all but one study (‘high’ risk; Parle *et al*^32^). Of the remaining eight studies, one found a significant difference in the mean age of the treatment groups (‘high’ risk; Dupuis *et al*^31^). Two studies included a mixture of participants with both acute and chronic tendinopathy (range of duration of symptoms 1–120 months), which may respond differently to treatment (‘high’ risk; Rio *et al*^35^ and van Ark *et al*^36^). Even though cross-over trials can sometimes be susceptible to carry-over effects, the cross-over design of two of the studies (Holden *et al*^22^ and Rio *et al*^20^) was considered unlikely to introduce bias as the participants only had one session of each intervention separated by an adequate time period. Adherence of participants to assigned treatment was low in the study by Dupuis *et al*^31^ and Clifford *et al*^17^ (‘high’ risk; table 2), while it was unclear in the studies by Parle *et al*,^32^ Stasinopoulos and Stasinopoulos,^33^ Rio *et al*,^35^ and van Ark *et al*^36^ (‘unclear risk’).

#### External validity

General, non-specific populations were used in all studies but four, which included athletes of specific sports (tennis, volleyball and basketball) and were therefore rated as ‘high’ risk as their findings cannot be generalised to the wider population (Rio *et al*,^20^ Rio *et al*,^35^ van Ark *et al*,^36^ and Stasinopoulos and Stasinopoulos^33^). In the remaining six studies, age ranges of participants were wide enough to allow for good generalisability. Clinically relevant assessment tools and outcome measures were used in all studies. The nature, frequency and intensity of treatments were considered appropriate in all studies.

### Precision

Statistical power calculation prior to recruitment was performed in only four studies, where their sample size was adequate for at least 80% power (Gatz *et al*,^37^ Holden *et al*,^22^ Dupuis *et al*^31^ and Vuvan *et al*^34^); all other studies were characterised as ‘high’ risk of precision bias. Levels of significance were set at p=0.05 in all studies.

### Findings of included studies

Tables 4 and 5 summarise the findings along with levels of evidence for the overall results of each outcome measure for studies. Tables 6 and 7 display the treatment effect for pain of isometric exercise versus control.

**Table 4:** Findings of studies that assessed outcomes immediately after exercise (45 min postintervention)

Treatment modes	Tendon affected	First author (year)	Pain	Functional disability	ROM	Strength	QoL	Structural integrity	Cortical inhibition
Isometric exercise versus isotonic exercise		Rio *(*2015)^20^	↓ (ΝRS)	–	–	↑	–	–	↑
	Patellar	Rio *(*2017)^35^	↓ (ΝRS)	–	–	–	–	–	–
		Holden (2020)^22^	↔ (NRS)	–	–	–	–	↔	–
Overall isometric versus isotonic exercise (evidence level)			↔ (3)	–	–	↑ (3)	–	↔ (3)	↑ (3)

↓: lower at statistical significance*; ↑: higher at statistical significance*; ↔: no statistically significant difference.

*With the first versus the second intervention.

NRS, Numeric Rating Scale; QoL, quality of life; ROM, range of movement.

**Table 5:** Findings of studies reporting short-term outcomes (up to 12 weeks of follow-up)

Acute/chronic tendinopathy	Treatment modes	Tendon affected	First author (year)	Pain	Functional disability	ROM	Strength	Satisfaction	Structural integrity	QoL
Acute	Combined isometric exercise/ice therapy versus ice therapy	Rotator cuff	Parle (2017)^32^	↔ (VAS)	↔ (DASH)	–	↔	–	↔	–
	Isometric exercise versus ice	Rotator cuff	Parle (2017)^32^	↔ (VAS)	↔ (DASH)	–	↔	–	↔	–
			Dupuis (2018)^31^	↔ (BPI)	↔ (DASH)	↔	↔	–		↔ (WORC)
	Overall isometric exercise versus ice (evidence level)			↔ (3)	↔ (3)	↔ (3)	↔ (3)	↔ (3)	↔ (3)	↔ (3)
Chronic	Isometric versus isotonic exercise	Patellar	van Ark (2016)^36^	↔ (NRS)	↔ (VISA-P)	–	–	↔ (GROC)	–	–
		Greater trochanteric pain syndrome	Clifford (2019)^17^	↔ (NRS)	↔ (VISA-G)	–	–	↔ (GROC)	–	↔ (EQ-5D-5L)
	Overall isometric versus isotonic exercise (evidence level)			**↔** (3)	**↔** (3)	–	–	**↔** (3)	–	**↔** (3)
Chronic	Combined isometric/isotonic exercise versus isotonic exercise	Wrist extensors	Stasinopoulos (2017)^33^	↓ (VAS)	↓ (pain-free grip strength)	–	–	–	–	–
		Achilles	Gatz (2020)^37^	–	↔ (VISA-A, AOFAS)	–	–	–	–	–
	Overall combined isometric/isotonic exercise versus isotonic exercise (evidence level)			**↓** (3)	**↔** (3)	–	–	–	–	–
	Isometric exercise versus no treatment	Wrist extensors	Vuvan (2020)^34^	–	↔ (pain-free grip strength) **↓** (PRTEE)	–	–	↔ (GROC)	–	–

↓: lower at statistical significance*; ↑: higher at statistical significance*; ↔: no statistically significant difference.

*With the first versus the second intervention.

AOFAS, American Orthopaedic Foot and Ankle Score; BPI, Brief Pain Inventory; DASH, Disabilities of the Arm, Shoulder and Hand; EQ-5D-5L, EuroQoL 5 Dimensions 5 Level Index; GROC, Global Rating of Change; NRS, Numeric Rating Scale; PRTEE, Patient-Rated Tennis Elbow Evaluation; QoL, quality of life; ROM, range of movement; VAS, Visual Analogue Scale; VISA-A, Victorian Institute of Sport Assessment-Achilles tendon; VISA-G, Victorian Institute of Sport Assessment-gluteal tendons; VISA-P, Victorian Institute of Sport Assessment-patellar tendon; WORC, Western Ontario Rotator Cuff Index.

**Table 6:** Mean values of pain scales and treatment effect for pain of isometric exercise versus control (isotonic exercise): studies assessing outcomes immediately after exercise (45 min postintervention)

Acute/chronic	Treatment modes	Tendon affected	First author (year)	Pain scale	Scale range	Isometric group pain score		Control group pain score		Mean treatment effect for pain (95% CI)	P value <0.05
						Baseline (1)	Postintervention (2)	Baseline (3)	Postintervention (4)		
Chronic	Isometric versus isotonic exercise	Patellar	Rio (2015)^20^	NRS (during SLDS)	0–10	7	0.2	6.3	3.8	−4.3 (−1.2 to −7.4)	Yes
			Rio (2017)^35^	NRS (during SLDS)	0–10	5	3.2	5	4.1	−0.9 (−1.1 to −0.7)	Yes
			Holden (2020)^22^	NRS (during SLDS)	0–10	5	4.2	4.3	3.2	+0.3 (1.3 to −0.7)	No

NRS, Numeric Rating Scale; SLDS, single leg decline squat.

**Table 7:** Mean values of pain scales and treatment effect for pain of isometric exercise versus control (isotonic exercise, ice or no treatment): studies assessing outcomes in the short-term (up to 12 weeks)

Acute/chronic	Treatment modes	Tendon affected	First author (year)	Pain scale	Scale range	Isometric group pain score		Control group pain score		Mean treatment effect for pain (2–1) – (4–3) (95% CI)	P value <0.05
						Baseline (1)	Longest follow-up (2)	Baseline (3)	Longest follow-up (4)		
Acute	Combined isometric exercise/ice therapy versus ice therapy	Rotator cuff	Parle (2017)^32^	VAS	0–10	4.8	3.7	5.5	4.5	−0.1 (N/A)	No
	Isometric exercise versus ice	Rotator cuff	Parle (2017)^32^	VAS	0–10	6.3	4.5	5.5	4.5	−0.8 (N/A)	No
			Dupuis (2018)^31^	BPI	0–10	3.2	1.6	2.7	1.2	−0.1 (−1.2 to 1)	No
Chronic	Isometric versus isotonic exercise	Patellar	van Ark (2016)^36^	NRS	0–10	6.3	4	5.5	2	−0.5* (−2.6 to 1.6)	No
		Greater trochanteric pain syndrome	Clifford (2019)^17^	NRS	0–10	5.9	3.9	5.9	3.2	+0.7 (−0.7 to 1.7)	No
Chronic	Combined isometric/isotonic exercise versus isotonic exercise	Wrist extensors	Stasinopoulos (2017)^33^	VAS	0–10	6.9	1.6	6.9	2.9	−1.3 (−0.8 to −1.9)	Yes

CIs have been calculated (see Statistical analysis section).

*Values at baseline and follow-up are median and not mean; therefore, the 0.5 value (also median) reported by the authors cannot be obtained from the calculation.

BPI, Brief Pain Inventory; N/A, not applicable; NRS, Numeric Rating Scale; VAS, Visual Analogue Scale.

### Lateral elbow tendinopathy

#### Isometric exercise versus no treatment

##### Short-term outcomes

One good-quality study compared (unsupervised) isometric exercise with no treatment for lateral elbow tendinopathy for 8 weeks.^34^ The isometric exercise group had a lower PRTEE score at 8 weeks compared with the ‘wait and see’ group, suggesting less functional disability. However, pain-free grip strength test, which we also classified as a test for ‘functional disability’, was similar between the two groups at 8 weeks. Similarly, GROC was also similar in the two groups at follow-up, even though 86% of participants in the isometric group reported an overall improvement versus 63% in the no treatment group (difference non-statistically significant). Pressure pain thresholds, heat pain thresholds and cold pain thresholds were also similar between the two groups at 8 weeks.

Overall, there is insufficient evidence for definitive conclusions on the short-term effectiveness of isometric exercise compared with no treatment in chronic lateral elbow tendinopathy. A single study of good overall quality (limited evidence; level 3) reported conflicting results with regard to functional disability and no difference in satisfaction.

#### Combined isometric/isotonic exercise versus isolated isotonic exercise

##### Short-term outcomes

One study of poor overall quality compared combined isometric plus eccentric-concentric exercise versus eccentric exercise versus eccentric-concentric exercise for 4 weeks in amateur tennis players with chronic lateral elbow tendinopathy.^33^ Within all three treatment groups, both pain (Visual Analogue Scale (VAS)) and functional disability (pain-free grip strength) improved significantly at 4 weeks and 8 weeks from the start of treatment compared with baseline. The improvement in the combined isometric/eccentric-concentric group was greater than the other two groups at both follow-up time points.

### Achilles tendinopathy

#### Combined isometric/isotonic exercise versus isolated isotonic exercise

##### Short-term outcomes

One study of poor overall quality compared combined isometric and isotonic (eccentric) exercise versus isolated isotonic (eccentric) exercise for 3 months in patients with chronic Achilles tendinopathy (Gatz *et al*^37^). No differences were found between the two groups at follow-up (1 and 3 months) in functional disability (VISA-A and American Orthopaedic Foot and Ankle Score (AOFAS)); however, the VISA-A improved significantly at 3 months compared with baseline in both groups and the AOFAS score in the isotonic-only group.

### Rotator cuff tendinopathy

#### Isometric exercise versus ice therapy

##### Short-term outcomes

One good-quality and one poor-quality study compared isometric exercise with ice therapy (cryotherapy) in patients with acute rotator cuff tendinopathy.

Parle *et al*^32^ randomised their participants to isometric exercise, ice therapy or a combination of the two for 1 week and found no between-group differences at 1-week follow-up with regard to pain (VAS), functional disability (DASH questionnaire), muscle strength or structural integrity (ultrasound scanning (USS)). All three groups demonstrated statistically significant improvements in all outcome measures at 1 week compared with baseline.

In the study by Dupuis *et al*^31^ participants were treated with either ice therapy or isometric exercise for 2 weeks and then both groups received isotonic exercise for a further 4 weeks. Even though both groups were found to have statistically significant improvements in pain (Brief Pain Inventory), strength, ROM, functional disability (DASH) and QoL (WORC) at 2-week and 6-week follow-up compared with baseline, there were no significant differences between the two groups at either time point.

### Patellar tendinopathy

#### Isometric exercise versus isotonic exercise

##### Immediate postintervention outcomes

One good-quality and two poor-quality studies compared immediate, postintervention effects of isometric and isotonic exercise in patellar tendinopathy following a single session of loading.

Rio *et al*^20^ performed a cross-over study of six jumping athletes with patellar tendinopathy (duration of symptoms not reported) comparing the two modes of exercise. All outcome measures (pain, strength and cortical inhibition) were recorded at baseline and immediately postintervention, with pain and strength also recorded 45 min postintervention. Pain (Numeric Rating Scale (NRS)) during a single leg decline squat (immediately postintervention) decreased significantly from baseline in both modes of exercise; however, the reduction was statistically greater in the isometric group. This reduction was sustained at 45 min in the isometric group but not in the isotonic group. Similarly, isometric exercise was associated with a statistically significant increase in strength (maximum voluntary isometric contraction torque) both immediately postintervention and at 45 min compared with baseline, which was not observed in the isotonic group. Finally, short-interval intracortical inhibition was found to be significantly higher (more favourable) postisometric exercise versus postisotonic exercise compared with baseline at statistical significance.

The same authors, using the same participants as their previous study,^35^ compared pain (NRS) during a single leg decline squat immediately after intervention in a group treated with isometric and a group treated with isotonic exercise. The mean reduction in pain immediately postintervention versus preintervention was significantly greater in the isometric group.

In a cross-over study by Holden *et al*,^22^ participants performed a single session of either isometric or dynamic isotonic exercise and outcome measures were recorded immediately postintervention and at 45 min. There were no differences in pain (NRS) during a single leg decline squat immediately postintervention or at 45 min compared with baseline with either isometric or dynamic exercise. There were no between-group differences at the two time points. Similarly, pressure point thresholds of the patellar tendon were similar at baseline, immediately postintervention and at 45 min without intergroup differences. Finally, there were no changes in patellar tendon thickness on USS before and after intervention with the two exercise modes.

### Patellar tendinopathy

#### Isometric exercise versus isotonic exercise

##### Short-term outcomes (≤12 weeks)

One poor-quality study compared short-term effects of isometric and isotonic exercise in chronic patellar tendinopathy. van Ark *et al*^36^ conducted a study in jumping athletes with patellar tendinopathy where participants received either an unsupervised isometric or isotonic exercise programme for 4 weeks. Although both groups improved at 4 weeks compared with baseline in terms of all pain (NRS), functional disability (VISA-P questionnaire) and satisfaction (GROC), no significant between-group differences were observed. Range of duration of symptoms was reported as 1–120 months (mean 35.8 months).

### Gluteal tendinopathy

#### Isometric exercise versus isotonic exercise

##### Short-term outcomes (≤12 weeks)

One poor-quality study assessed the short-term benefits of isometric versus isotonic exercise in chronic gluteal tendinopathy. Clifford *et al*^17^ randomised patients with greater trochanteric pain syndrome (GTPS) to either isometric or isotonic exercise (both unsupervised) for 12 weeks. In this pilot RCT, descriptive statistics suggested there were no observed differences between the two groups at either 4-week or 12-week follow-up even though p values were not used. Both groups had similar improvements in functional disability (VISA-G), pain (NRS) and satisfaction (GROC) at both follow-up time points compared with baseline. The remainder of outcome measures (Pain Catastrophising Scale, Hip Disability and Osteoarthritis Outcome Score (HOOS), EuroQoL 5 Dimensions 5 Level Index, and International Physical Activity Questionnaire-Short Form) were also similar between groups at both time points with minimal changes between baseline and 12 weeks. The only statistically significant benefits were observed between baseline and 12 weeks in the pain and QoL subcomponents of the HOOS questionnaire in the isotonic group.

### Pooled results

Where two or more studies compared the same interventions at similar follow-up time points, their results were combined qualitatively based on direction of effect to make conclusions on the effectiveness of interventions.

#### Isometric exercise versus ice therapy

Overall, based on limited evidence (level 3), isometric exercise is not associated with short-term benefits in pain, functional disability, ROM, strength, QoL and structural integrity compared with ice therapy in acute rotator cuff tendinopathy.

#### Isometric exercise versus isotonic exercise

Based on limited evidence (level 3), immediate postintervention pain, pressure point thresholds and tendon structural integrity appear to be similar with isometric and isotonic exercise in patellar tendinopathy. Based on a single study of good quality, there may be no immediate postintervention benefits in pain with either isometric or isotonic exercise. Compared with isotonic exercise, isometric exercise may be associated with increased strength and cortical inhibition immediately after exercise; however, this is based on a single study of poor quality.^20^ We emphasise that the results of all three studies are based on assessment before and immediately following exercise sessions.

**Figure 2:**
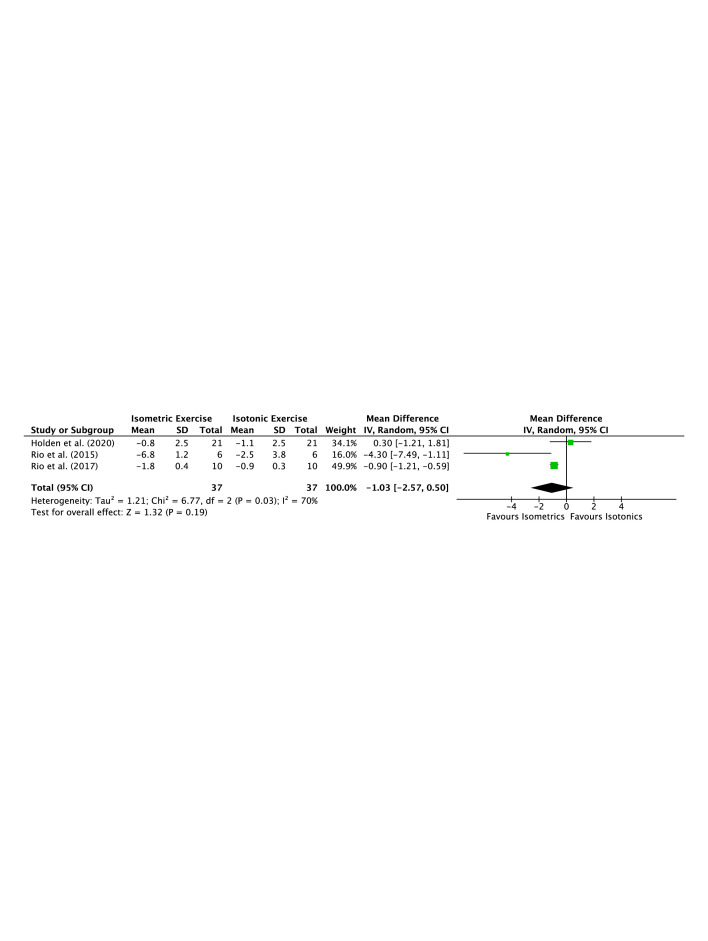
Forest plot for the comparison between isometric and isotonic exercise with regard to immediate postintervention improvement in reported pain. IV, intervention.

Figure 2 illustrates a forest plot for the comparison between isometric and isotonic exercise with regard to the immediate postintervention improvement in reported pain. Statistical analysis showed no significant difference between the two interventions (p=0.19), which reinforces our aforementioned qualitative conclusion.

With regard to short-term follow-up, based on limited evidence (level 3), isometric and isotonic exercises appear to be similar in terms of their benefits in pain, functional disability, satisfaction and QoL in chronic tendinopathy.

#### Combined isometric/isotonic exercise versus isolated isotonic exercise

Based on two studies of poor quality (limited evidence; level 3), combined isometric plus isotonic exercise may be superior to isolated isotonic exercise in the short term for pain but not for functional disability (conflicting evidence). This conclusion however may be biased due to the different types of isotonic exercise used (eccentric only vs concentric/eccentric) as control in the two studies.

Furthermore, we do recognise that the heterogeneity in the last two grouped comparisons in terms of tendinopathy location (patellar vs gluteal and lateral elbow vs Achilles) in study participants is an important limitation and these findings should be interpreted with caution.

## Discussion

This systematic review found that isometric exercise was not superior to isotonic exercise in terms of pain in chronic tendinopathy either immediately after a single session or in the short term (follow-up ≤12 weeks). These findings are based on limited evidence (level 3) and they arise from patients with tendinopathies of different sites, except for the conclusion from immediate postintervention outcomes which are specific to patellar tendinopathy. Analysis of secondary outcomes also failed to demonstrate any significant differences either immediately or short term. Additionally, we found no significant short-term benefits of isometric exercise compared with ice therapy for acute rotator cuff tendinopathy with regard to any of our primary or secondary outcome measures (limited evidence; level 3).

Three studies have investigated the immediate effect of both isometric exercise and isotonic exercise for pain in patellar tendinopathy with variable results. Rio *et al*^20^ reported a significant reduction in pain following isometric exercise (mean=6.8 points), with smaller reductions observed with isotonic exercise (mean=2.5 points) when performing a single leg decline squat. Both groups demonstrated improvement greater than the clinically important difference of 2 points.^38^ A subsequent study by Rio *et al*^35^ in jumping athletes found that isometric exercise was more effective at reducing pain than isotonic exercise (mean=1.8 vs 0.9 points). Holden *et al*^22^ reported pain reduction for both isometric exercise (mean=0.8 points) and isotonic exercise (mean=1.1 points) in a study in which the methodology was almost identical to the study by Rio and colleagues^20^ but with a larger population. Pearson *et al*^23^ compared two different isometric loading protocols for patellar tendinopathy (10 s and 40 s holds) and an immediate reduction in pain (mean=1.7 points) was reported for both groups. Two observational studies for plantar fasciopathy and Achilles tendinopathy both used a similar isometric loading protocol to Rio *et al*.^20^ However, the immediate pain response was found to be variable in both studies. Isometric exercise was not superior to either isotonic exercise or walking in plantar fasciopathy, with only 15% of participants reporting a clinically meaningful pain reduction following isometric loading.^24^ For Achilles tendinopathy, 45 s isometric holds of the ankle plantar flexors resulted in reductions in pain of 1 point in some participants, with others reporting an immediate increase in pain.^25^ Taken together, there is conflicting evidence that isometric exercise provides significant, immediate pain relief in chronic tendinopathy. The large pain reductions observed in a single study of six male volleyball players with patellar tendinopathy have not been replicated and therefore may not be generalisable to other tendinopathy populations.

We examined the short-term effects (≤12 weeks) of isometric exercise to either another treatment or no treatment. Overall, isometric exercise was found to be effective in providing pain relief and improving functional disability in tendinopathy, but there is no evidence that it is superior to isotonic exercise. Clifford *et al*^17^ compared isometric exercise with isotonic exercise for GTPS and found no difference between groups at either 4 or 12 weeks. van Ark *et al*^36^ also reported no difference between isometric and isotonic exercise after 4 weeks in patellar tendinopathy. In both studies, the volume of loading or time under tension (TUT) was identical for each group for the duration of the intervention. Given that no difference was found between isometric and isotonic loading after 4 or 12 weeks, muscle contraction type may be less important when TUT is equal as the tendon appears to respond in a similar manner.^19 39^ Furthermore, it suggests that isometric exercise can also be used for progressive tendon loading and not only for acute pain relief as has previously been proposed.^21^

In lateral elbow tendinopathy a combined programme (isometric plus eccentric-concentric exercise) was more effective after 4 weeks than either an eccentric programme or an eccentric-concentric programme.^33^ The combined programme consisted of 56 min of loading per session compared with 22 min for the other two programmes. Gatz *et al*^37^ compared eccentric exercise with eccentric exercise combined with isometric exercise for Achilles tendinopathy. No additional benefit was observed with the addition of isometric exercise after either 1 or 3 months. This appears surprising as the TUT was higher in the combined group. A possible explanation for the differences between both studies relates to the loading intensity. For the lateral elbow, progressive loading was achieved by adding weights. However, for the Achilles no external weight was used, and load was progressed in both groups using bodyweight, that is, bilateral to unilateral loading. Progressive tendon loading appears to be critical in the management of tendinopathy, and while this may be achieved by increasing TUT it needs to be considered in conjunction with intensity.

The mechanism by which loading provides pain relief in tendinopathy is not yet fully understood, reflecting the complex multifactorial nature of tendon disease. Exercise-induced hypoalgesia (EIH) occurs in response to exercise, including isometric exercise, in healthy populations and is believed to occur via a number of pathways including descending pain inhibition.^40 41^ Approximately 35%–45% of patients with tendinopathy do not make significant improvements with loading programmes,^15–17^ and the reasons for this are largely unknown. EIH is not present in some individuals with chronic musculoskeletal pain,^42^ although we are not aware of any studies that have measured this in tendinopathy. Isometric exercise has been found to increase pain in some chronic pain populations,^43^ and this may be partly due to the presence of central sensitisation (a physiological phenomenon characterised by widespread hypersensitivity resulting from an augmented response of central neurons to receptor activity). Central sensitisation can also be a feature of tendinopathy,^44 45^ and when present may explain why some individuals experience an increase in pain with isometric and isotonic loading. This hypothesis would possibly be supported by the findings of Coombes *et al*^46^ in lateral elbow tendinopathy. One of the recent International Scientific Tendinopathy Symposium (ICON) consensus statement, authored by international tendinopathy experts, recommended measuring central sensitisation in future tendinopathy research as it may be useful in subgrouping studies.^47^ A further ICON consensus statement highlighted that patient characteristics relating to general health may be a major confounder to treatment outcomes in tendinopathy.^48^ Patients with chronic tendinopathy, especially older and more sedentary individuals, often have associated comorbidities, for example, diabetes,^49^ high cholesterol^50^ and high body mass index.^51^ These characteristics are not routinely measured in tendinopathy studies, but it is recommended that they are reported in future clinical trials. The presence of these characteristics either independently or in combination may be associated with a poorer response to loading programmes and a poorer treatment outcome. Future research identifying which characteristics are more likely to affect treatment outcome and response to loading programmes will be beneficial.

### Limitations

Despite the inclusion of all relevant studies in the literature and the detailed quality assessment performed, we recognise the limitations of our systematic review. First, the majority of studies did not include a control group that received no treatment; therefore, the effect of time (natural healing) and its contribution to the improvement in outcome measures observed with the different exercise regimens could not be assessed. Additionally, due to the small number of eligible studies, our results were only based on limited evidence and were generalised to all types of tendinopathy with the assumption that they all share the same underlying pathophysiology and respond similarly to the same types of loading. Finally, the lack of homogeneity in loading regimens, follow-up time points and outcome measures precluded the conduct of quantitative analyses for the majority of comparisons; however, a meta-analysis was conducted where it was appropriate.

## Conclusion

To our knowledge this is the first systematic review to investigate the effectiveness of isometric exercise in the management of tendinopathy. We found no strong evidence that isometric exercise is superior for immediate or short-term pain relief when compared with isotonic exercise, other treatments or no treatment. The response to isometric exercise appears to be variable both within and across tendinopathy populations. However, well-designed RCTs with larger sample sizes and long-term follow-up are needed.
